# TRAIL Receptor Signaling Regulation of Chemosensitivity In Vivo but Not In Vitro

**DOI:** 10.1371/journal.pone.0014527

**Published:** 2011-01-14

**Authors:** Christina Menke, Tatiana Goncharov, Lubna Qamar, Christopher Korch, Heide L. Ford, Kian Behbakht, Andrew Thorburn

**Affiliations:** 1 Department of Pharmacology, University of Colorado School of Medicine, Aurora, Colorado, United States of America; 2 Department of Obstetrics and Gynecology, University of Colorado School of Medicine, Aurora, Colorado, United States of America; 3 Department of Medicine, University of Colorado School of Medicine, Aurora, Colorado, United States of America; National Cancer Institute, United States of America

## Abstract

**Background:**

Signaling by Tumor Necrosis Factor-Related Apoptosis Inducing Ligand (TRAIL) and Fas ligand (FasL) has been proposed to contribute to the chemosensitivity of tumor cells treated with various other anti-cancer agents. However, the importance of these effects and whether there are differences in vitro and in vivo is unclear.

**Methodology/Principal Findings:**

To assess the relative contribution of death receptor pathways to this sensitivity and to determine whether these effects are intrinsic to the tumor cells, we compared the chemosensitivity of isogenic BJAB human lymphoma cells where Fas and TRAIL receptors or just TRAIL receptors were inhibited using mutants of the adaptor protein FADD or by altering the expression of the homeobox transcription factor Six1. Inhibition of TRAIL receptors did not affect in vitro tumor cell killing by various anti-cancer agents indicating that chemosensitivity is not significantly affected by the tumor cell-intrinsic activation of death receptor signaling. However, selective inhibition of TRAIL receptor signaling caused reduced tumor regression and clearance in vivo when tested in a NOD/SCID mouse model.

**Conclusions:**

These data show that TRAIL receptor signaling in tumor cells can determine chemosensitivity in vivo but not in vitro and thus imply that TRAIL resistance makes tumors less susceptible to conventional cytotoxic anti-cancer drugs as well as drugs that directly target the TRAIL receptors.

## Introduction

The death receptors DR4 and DR5 activate signaling and apoptosis in response to the Tumor Necrosis factor-Related Apoptosis-Inducing Ligand (TRAIL), while Fas/CD95 activates apoptosis in response to Fas ligand (FasL). These receptors are the main executioners of the “extrinsic” apoptosis pathway that activate the apoptosis machinery by forming a complex called the Death Inducing Signaling Complex (DISC). The DISC is formed when a ligand-bound receptor complex recruits the adaptor protein FADD, which leads to the recruitment, dimerization [1], and catalytic activation of caspase-8 [2]–[4]. Active caspase-8 directly activates the effector caspase-3 and stimulates the mitochondrial (intrinsic) apoptosis pathway by cleaving the BH3 protein Bid. This allows Bid's translocation in to the mitochondria and Bax/Bak-dependent release of cytochrome c and other pro-apoptotic proteins, with subsequent amplification of effector caspase activity. There is considerable interest in targeting the TRAIL receptors using pro-apoptotic receptor agonists [2] and clinical trials using recombinant TRAIL and antibodies that target DR5 or DR4 are underway.

The TRAIL and Fas pathways are important in anti-tumor and anti-metastasis responses mediated through the immune system [3], [5]. TRAIL signaling mediates T-cell- and natural killer (NK) cell-dependent metastasis suppression in xenografts [6]–[9]. Autochthonous models show that deficiency in TRAIL receptor signaling promotes tumorigenesis [10] and metastasis [11]. Fas signaling has also been proposed as a mechanism by which NK cells can eliminate tumor cells [12]. Conversely, Fas signaling can also be a mechanism by which tumors counteract immune-mediated anti-tumor responses [13]. Moreover, both Fas [14] and TRAIL [15] have non-apoptotic signaling activities that promote tumor progression if the apoptotic response is blocked. Tumor cells can become resistant to death receptor signaling through multiple mechanisms [16]. Some of these mechanisms e.g. down-regulation of FADD [17] or increased expression of the caspase-8-like protein FLIP [18] affect both Fas and TRAIL receptors whereas, other mechanisms are more selective. For example, somatic mutations in DR5 cause a dominant negative phenotype that blocks TRAIL signaling through DR4 and DR5, but has no effect on Fas signaling [19]. Similarly, increased expression of the homeobox transcription factor Six1 is a common tumor defect that arises in the majority of patients with metastatic ovarian or breast cancer, is associated with poor clinical outcome in multiple tumor types [20] and causes inhibition of TRAIL but not FasL-induced apoptosis [21].

Most anti-cancer drugs function by activating the mitochondrial apoptosis pathway; however, it has been suggested that death receptor signaling also contributes to the overall anti-tumor response to diverse chemotherapeutic drugs. Drug and radiation induced killing of brain tumor [22] and hepatoma [23] cells have been reported to rely on Fas signaling. Experiments, where TRAIL signaling was inhibited by silencing DR5 [24] or by increasing the expression of the decoy receptor DcR2 [25], led to the conclusion that chemosensitivity to 5-fluorouracil, doxorubicin, and etoposide depends on TRAIL receptor signaling. These effects have been demonstrated in vitro with cell lines, suggesting they are intrinsic to tumor cells. These effects can also be achieved by increased expression of death receptors and/or ligands that create a tumor cell-intrinsic autocrine signaling loop. Similar mechanisms of death receptor up-regulation have been proposed as an explanation for how various cytotoxic chemotherapeutic agents synergize TRAIL receptor-targeted agonists [26].

However, it is unclear if the Fas and TRAIL receptor pathways are really important contributors to tumor chemosensitivity. Since the activation of the mitochondrial apoptosis pathway leads to efficient cell killing, one would expect that drugs that are able to activate the mitochondrial pathway (i.e., most anti-cancer agents) should not require additional death receptor signaling in order to die, unless the pro-apoptotic signal from the mitochondria was insufficient to force the cell to cross its apoptotic threshold. It is less clear if the same considerations apply in vivo, where other inputs (e.g., from other cell types) may play a role. To address this question, we constructed isogenic tumor cell lines that are functional for both TRAIL and FasL signaling, inhibited for both or inhibited for just TRAIL signaling. We show that even in a cell line in which blocking death receptor-induced apoptosis has no detectable effect on the sensitivity to various chemotherapeutic agents and other apoptotic inducers in vitro, inhibition of TRAIL receptor signaling in vivo affects sensitivity to an anti-cancer drug. These data indicate that the presence of a functional TRAIL receptor apoptosis pathway can regulate chemosensitivity through tumor cell extrinsic mechanisms.

## Results and Discussion

### Selective inhibition of death receptor signaling with FADD-DD mutants

**Figure 1:**
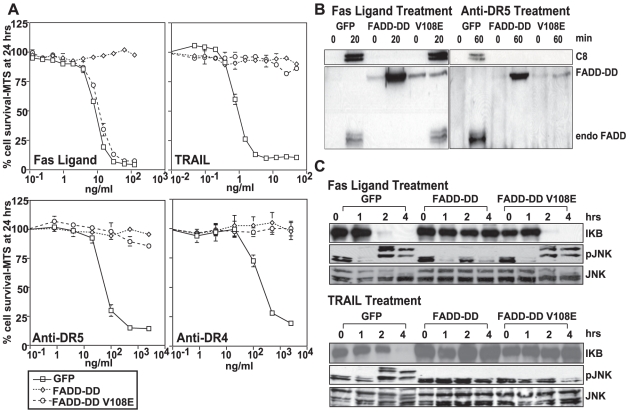
Selective inhibition of FasL and TRAIL or TRAIL-induced signaling and apoptosis. In panel A, isogenic BJAB cells expressing GFP or GFP-FADD-DD and FADD-DDV108E were treated with increasing doses of Fas ligand, TRAIL, or agonistic anti-DR4 and anti-DR5 antibodies. The differences in responses indicate that both FADD-DD expressing cell lines were resistant to all TRAIL R targeted drugs, but that only FADD-DD expressing cells are resistant to FasL. In panel B, DISC IP experiments precipitating Fas or DR5 followed by western blotting for casp-8 or FADD demonstrate an increased recruitment of FADD-DD in place of endogenous FADD to the receptors. Panel C, illustrates that on activation of other signaling pathways leading to IkB degradation, and JNK phosphorylation, FADD-DD blocks signaling. This block leads to these events for both FasL and TRAIL, and that FADD-DD V108E blocks signaling only for TRAIL.

FADD is required for both TRAIL- and FasL-induced apoptosis. One way signaling can be inhibited by these receptors is by overexpressing a version of FADD (FADD-DD) that contains the FADD death domain, but lacks the death effector domain that binds to caspase-8. This molecule has been thought to inhibit signaling by competing with endogenous FADD protein for binding to the activated death receptors. However, based on data showing that FADD must self-associate via its death effector domain in order to bind to death receptors, it has been proposed that the isolated FADD death domain should be unable to bind to or efficiently inhibit Fas signaling [27]. Therefore, we first tested if we could obtain effective and selective inhibition of death receptor-induced apoptosis using FADD-DD and FADD-DD V108E, a mutant that was selected for its inability to bind to Fas, while retaining the ability to bind to TRAIL receptors [28]. Dose response curves (Figure 1A) using FasL or TRAIL with three isogenic BJAB cell lines expressing, GFP, GFP-FADD-DD or GFP-FADD-DD (V108E) showed that FADD-DD and FADD-DD (V108E) effectively inhibited apoptosis induced by TRAIL and agonistic TRAIL receptor antibodies. However, only the wildtype FADD-DD molecule inhibited FasL-induced death.

To test if inhibition of receptor-induced apoptosis was due to binding of the FADD-DD molecules to the activated receptors, we performed DISC immunoprecipitation experiments (Fig. 1B). Upon activation of the receptor, FADD-DD was recruited to both Fas and TRAIL receptors instead of the endogenous FADD protein, which was recruited in the control cells. The V108E mutant was recruited only to activated TRAIL receptors. These data indicate that FADD-DD molecules are effective inhibitors of death receptor signaling and that their mechanism of action is through recruitment to the activated receptor in place of endogenous FADD protein. However, because the level of the FADD-DD mutants (Fig. 1A) in the cells is about 200-fold higher than the endogenous FADD protein, while the amount of the FADD-DD recruited to activated receptors is similar to the amount of endogenous FADD that is recruited, our data are consistent with the conclusion of Sandu et al. [27] that the isolated death domain is less efficiently recruited to the receptors compared with the endogenous protein. Fig. 1C demonstrates that the FADD-DD molecule also blocks both FasL and TRAIL-induced activation of downstream kinase pathways activating JNK and causes degradation of IκB. The V108E mutant only affects TRAIL-induced activation of these pathways, which are known to be activated in a FADD-dependent manner [29].

### Inhibition of Fas and TRAIL receptor-induced apoptosis has no effect on the efficiency of tumor cell killing by diverse chemotherapeutic agents and apoptotic stimuli in vitro

**Figure 2:**
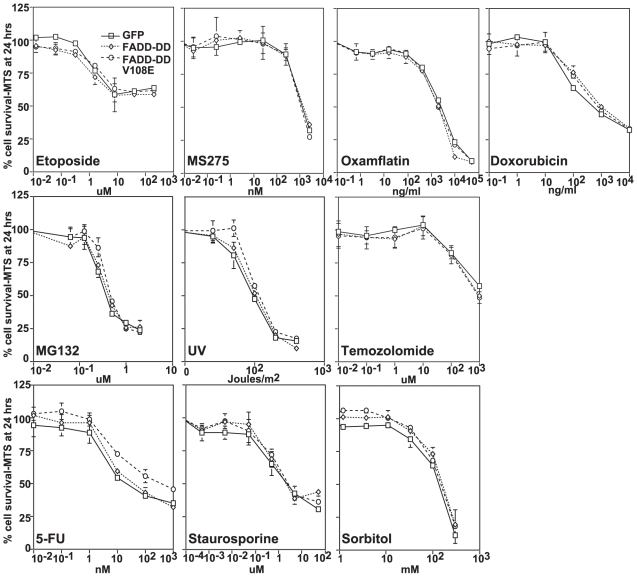
FADD-DD and FADD-DD V108E do not inhibit killing by other apoptotic stimuli. Isogenic BJAB cell lines were treated with increasing doses of etoposide, MS-275, oxamflatin, doxorubicin, MG132, UV, temozolomide, 5-FU, staurosporine or sorbitol as indicated followed by MTT assay to assess cell viability. All dose response curves overlap for each stimulus.

**Figure 3:**
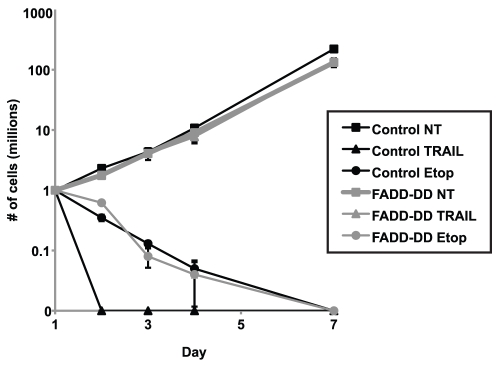
FADD-DD blocks TRAIL-induced but not etoposide-induced death in long-term assays. Isogenic control or FADD-DD expressing BJAB cells were treated with TRAIL or etoposide as indicated for 24 hours, then washed and replaced into growth media. Long term growth of surviving cells was determined by counting viable cells. Control BJAB cells died rapidly and were unable to recover any long term growth. Etoposide treated cells were completely unable to recover growth capacity whether or not FADD-DD was expressed. However FADD-DD expression protected the TRAIL-treated cells as demonstrated by overlapping growth curves with the untreated controls.

To test whether death receptor signaling alters the sensitivity of tumor cells to other agents, we assessed dose response curves for the three isogenic cell lines with agents that work by different mechanisms. Overlapping dose response curves (Fig. 2) showed that they had no measurable effect on tumor cell killing by various types of agents that target activities that are relevant for anti-cancer treatment. We observed this for a toposiomerase inhibitor (etoposide), histone deactylase inhibitors (oxamflatin, MS275), an anthracycline (doxorubicin), a proteosome inhibitor (MG132), DNA damaging agents (UV, temozolomide) and an antimetabolite (5-fluorouracil). Similarly, there was no effect of FADD-DD or the V108E mutant on tumor cell killing by general apoptotic stimuli including the broad-spectrum protein kinase inhibitor staurosporine and increased hyperosmolar stress (sorbitol). MTS assays assess cell viability over a relatively short term and thus are not truly comparable to long-term tumor growth responses in vivo. To ensure that the selective inhibition of TRAIL-induced death without affecting survival in response to cytotoxic chemotherapy affected long-term growth, we performed a cell grow back assay by treating cells for 24 hours with TRAIL or etoposide then washing out the drug and allowing any surviving cells to grow back. Figure 3 shows that the TRAIL-treated FADD-DD expressing cells displayed equivalent growth over 7 days to untreated cells whereas the same cells died in response to etoposide treatment. Thus even with a more rigorous tumor cell survival assay where any surviving cells had several days to recover and grow in the absence of drug, FADD-DD provides no protection against etoposide-induced death, while providing complete protection against TRAIL.

**Figure 4:**
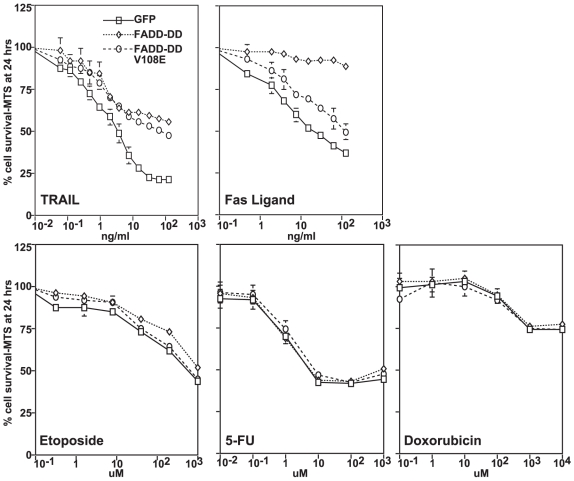
FADD-DD and FADD-DD V108E do not inhibit chemotherapy-induced death in HCT-116 cells. HCT-116 cells transfected with GFP, FADD-DD or FADD-DDV108E expression constructs were treated with increasing doses of TRAIL, FasL, etoposide, 5-FU, or doxorubicin as indicated and cell viability was assessed. FADD-DD and FADD-DDV108E inhibited FasL and TRAIL as in BJAB cells, but had no effect on tumor cell killing by the chemotherapy drugs.

These data run counter to some other studies. For example, Liu et al. [25] concluded that increased expression of DcR2, which is a decoy receptor that selectively inhibits TRAIL signaling, reduced in vitro chemosensitivity to doxorubicin and etoposide, while Wang and El-Deiry concluded that knockdown of the TRAIL receptor DR5 conferred resistance to 5-fluorouracil [24]. We therefore repeated our studies in a colon cancer cell line that was used by other investigators who reported effects on chemosensitivity. Figure 4 shows that HCT116 cells expressing FADD-DD were resistant to both TRAIL and FasL, while the FADD-DD (V108E) expressing cells were resistant only to TRAIL. However, neither of these cell lines displayed significantly increased resistance to 5-fluorouracil, etoposide, or doxorubicin. Additionally, we determined whether combination treatments with TRAIL and other anti-cancer agents demonstrated a requirement for death receptor signaling for optimal activity of the other drug. Combination treatments using TRAIL with 5-FU, Doxorubicin, or etoposide all showed increased tumor killing compared with treatment with the cytotoxic agent alone. However FADD-DD or FADD-DDV108E expression only blocked the component of the death due to the death receptor agonist (data not shown). These data indicate that tumor cell intrinsic signaling through the Fas and TRAIL receptors does not significantly contribute to the killing activity of the other stimuli in BJAB cells or in HCT-116 cells.

### Inhibition of TRAIL receptor-induced apoptosis promotes tumor growth and confers chemoresistance in vivo

**Figure 5:**
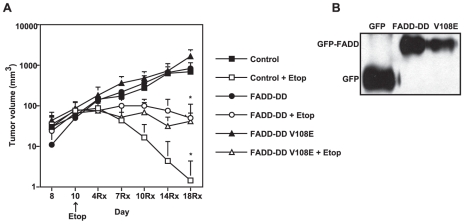
FADD-DD and FADD-DDV108E reduce the effectiveness of tumor eradication by etoposide in vivo. Panel A, isogenic BJAB cells expressing GFP control, GFP-FADD-DD and GFP-FADD-DDV108E were implanted subcutaneously and tumors grown for 10 days prior to treatment with etoposide. Untreated tumors continued to grow. In control BJAB cells, etoposide caused tumor eradication; whereas, in tumors expressing either FADD-DD or FADD-DDV108E, etoposide treatment led to stabilization of tumor mass but no eradication (p<0.05 by t-test at 18 for the control versus FADD-DD and FADD-DDV108E expressing cells). Panel B, Western blot of tumor tissue from GFP control, GFP-FADD-DD and GFP-VFADD-DD V108E demonstrating similar expression of the GFP-tagged protein in all tumors.

We next tested whether the FADD-DD constructs conferred an effect in vivo by growing xenograft tumors with each of the isogenic BJAB cell lines and treating with one of the agents (etoposide) that had no effect in vitro. Figure 5 shows that etoposide treatment caused almost complete tumor regression for the wildtype BJAB cells; whereas, the cells expressing FADD-DD or FADD-DD (V108E) displayed significantly less tumor regression (p<0.05) by etoposide. These data indicate that the same tumor cells whose sensitivity to etoposide is not affected by FADD-DD or the V108E mutant in vitro (in both short- and long- term assays), do display reduced chemosensitivity in vivo. Western blotting of tumor tissue showed that the tumors retained similar levels of expression of the GFP-tagged proteins in each case. This shows that the death receptor-dependent aspect of etoposide function is achieved through a tumor cell extrinsic mechanism. Because the FADD-DD and FADD-DD V108E mutants were equally effective at blocking tumor regression caused by etoposide, we conclude that signaling through TRAIL receptors alone is sufficient to cause these effects.

**Figure 6:**
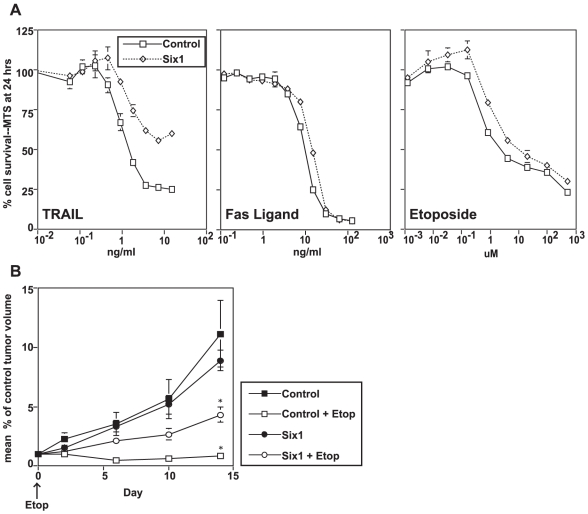
Six1-mediated TRAIL resistance reduces the effectiveness of etoposide in vivo, but not in vitro. Panel A, parental BJAB cells and BJAB cells expressing Six1 were treated in vitro with increasing doses of TRAIL, FasL and etoposide, and cell viability was assessed by the MTT assay. Six1 caused TRAIL resistance but had little effect on FasL or etoposide-induced cell death. Panel B illustrates how etoposide treatment of subcutaneous tumors from BJAB or BJAB-Six1 cells reduces the growth of both tumors relative to untreated controls, but is less effective in the Six1-expressing cells (* p<0.001 by t-test at day 14 for the control versus Six1 expressing cells).

The FADD-DD mutant is a useful tool, because it is highly specific and effective; however, such dominant negatives have not been found in human tumors. Therefore, to test whether these effects also apply when tumor cells are resistant to TRAIL through a mechanism that is relevant in human tumors, we compared isogenic BJAB cells that do or do not express the homeobox transcription factor Six1. Previously, we have shown that Six1 confers TRAIL resistance, but has little effect on FasL sensitivity [21]. Six1-expressing BJAB cells are TRAIL-resistant (Fig. 6A) to a somewhat lesser degree than FADD-DD expressing cells (Fig. 1). However, these Six1-expressing cells do not demonstrate altered FasL or etoposide sensitivity in vitro. Furthermore, these cells displayed reduced chemosensitivity to etoposide (p<0.05) when tested in vivo (Fig. 6B). Thus, the in vivo dependence of TRAIL receptor signaling for maximal chemosensitivity to another drug like etoposide also applies for a TRAIL resistance mechanism that is commonly found in human tumors and associated with poor clinical outcomes.

### Conclusions

Using isogenic tumor cells that differ only in their ability to undergo apoptosis in response to Fas or TRAIL receptor activation, we found that various anti-cancer agents display no significant difference in their ability to be killed in vitro by anti-cancer drugs. This shows that, in general, cancer chemotherapy drugs do not need to work through the death receptors. However, our results demonstrate a quite different and surprising result in vivo; etoposide, which was unaffected in vitro by TRAIL receptor or TRAIL and Fas receptor inhibition was significantly less effective in vivo and was unable to cause regression of these tumors. Instead, treatment led to stable tumor size when death receptor signaling was inhibited in the tumor cells. The lack of correlation between the in vitro and in vivo experiments carried out with the same cells indicates that even in tumor cells where activation of TRAIL receptors is not an important component of tumor cell killing in response to chemotherapy, tumor regression and clearance after treatment with a DNA damaging agent requires TRAIL receptor signaling.

This work suggests that tumors, which have evolved TRAIL resistance mechanisms [16] such as Six1 overexpression will not only respond less well to drugs such as Apo2L/TRAIL, lexatumumab, mapatumumab, ApoMab, AMG 655 etc. [2] that directly activate TRAIL receptors, but may also respond less well in vivo to conventional cytotoxic chemotherapy. The tumors were grown in NOD/SCID mice that lack T cells, but not Natural Killer cells or macrophages, suggesting that these cells are most likely the source of the TRAIL signal. Recent work has demonstrated the importance of the adaptive immune system, especially T cells, to the overall effectiveness of cancer chemotherapy (for review see [30]). Our data suggest that immune cell-mediated mechanisms working through TRAIL contribute to efficient tumor clearance after cytotoxic chemotherapy even without T cell involvement and these effects may add to any T cell mediated tumor clearance occurring after chemotherapy treatment. These data suggest that efforts to bypass TRAIL resistance would improve the efficacy of chemotherapy as well as improving the usefulness of drugs that are specifically targeted to TRAIL receptors.

## Materials and Methods

### Ethics Statement

This study was carried out in strict accordance with the recommendations in the Guide for the Care and Use of Laboratory Animals of the National Institutes of Health. The protocol was approved by the Animal Care and Use Committee of the University of Colorado, Anschutz Medical Campus (protocol # 72609(12)1E).

### Cell Lines

Parental BJAB cells were described previously [19], the various resistant cells expressing FADD-DD and FADD-DD V108E were made by stably expressing the respective cDNAs in pcDNA3.1 puro(+)-GFP. Six1-expressing cells were made by stably expressing the cDNA in pcDNA3.1 puro (+). All cell clones were derived from representative single clones isolated by limiting dilution. Cells were grown in RPMI 1640 with 10% FBS, sodium bicarbonate, and glucose in a 5% CO2 humidified atmosphere at 37°C. The BJAB cells used in these studies were DNA profiled using the Identifiler kit (Applied Biosystems) in January 2010. We have not found any publication of a DNA profile for BJAB cells, nor are there any profiles for these cells or ones that have a matching profile in our own database (CK) or in publicly available databases. These include the consolidation of the DSMZ, ATCC, JCRB, and Riken databases of DNA profiles of cell lines now available at DSMZ website (www.DSMZ.de). Therefore, it is impossible to compare our sample of these cells to samples of this line used in other reports. However, this analysis did exclude contamination with any common cell lines that have been previously profiled. The profile we obtained for these cells is the following: Amelogenin: X; CSF1PO: 8,10; D2S1338: 18, 21; D3S1358: 16; D5S818: 12, 13; D7S820: 10, 11;D8S1179: 14, 15; D13S317: 9,11; D16S539:9, 11; D18S51: 16, 22; D19S433: 12, 14; D21S11: 27, 28; FGA: 27, 28; THO1: 7; TPOX: 6,9; vWA: 14, 16.

### DISC IP

Fas Ligand—2.5×10^7^ cells were suspended in 25 ml of culture medium, incubated with SuperFasLigand (Enzo Life Sciences, Plymouth Meeting, PA) at 1.25 µg/ml at 37°C for 20 min, washed in phosphate-buffered saline three times, and then lysed in IP buffer (150 mM NaCl, 25 mM Tris·Cl, pH 7.5/1% Triton X-100, 4 mM EDTA) supplemented with complete protease inhibitors (Roche Applied Science) for 1 hr at 4°C. After the lysates were centrifuged (15 min at 13,000 rpm), lysates were precleared for 1 hr at 4°C with Glutathione-Agarose beads (Sigma, St. Louis, MO). Anti-Flag M2 beads (Sigma, St. Louis, MO) were added and lysates were incubated at 4°C overnight. The beads were washed six times with IP buffer and Flag Peptide (Sigma, St. Louis, MO) was added at 200 ug/ml. Samples were eluted at room temperature and concentrated. Samples were then subjected to Western blotting analysis. Anti-DR5—TR2J (Human Genome Sciences) was crosslinked with anti-human IgG Fc (Sigma, St. Louis, MO) in a 1∶1 ratio for 30 minutes prior to incubation with cells. Cells (2.5×10^7^) were suspended in 25 ml of culture medium, incubated with TR2J/IgG at 1 µg/ml at 4°C for 30 min, transferred to 37°C for another 1 hr, washed in phosphate-buffered saline three times, and then lysed in IP buffer for 1 hr at 4°C. After the lysates were centrifuged, lysates were precipitated at 4°C overnight. The beads were washed six times with IP buffer supplemented with 0.5 M NaCl and samples were subjected to Western blotting analysis.

### Cell death assays

BJAB cells were plated in 96 well plates at 40,000 cells per well. TR2J (anti-DR5) and Mapatumumab (anti-DR4) both provided by Human Genome Sciences were cross-linked with anti-human IgG Fc for 30 min prior to serial dilution. The following drugs were prepared according to manufacturer's instructions and were applied in serial dilution format: TRAIL (R&D Systems, Minneapolis, MN), SuperFas Ligand (Enzo Life Sciences, Plymouth Meeting, PA), 5-Fluorouracil (5-FU), Doxorubicin Hydrochloride, Etoposide, Oxamflatin, Temozolomide, Sorbitol, MS-275, and Staurosporine (Sigma, St. Louis, MO), MG132 (EMD Biosciences, Gibbstown, NJ). UV irradiation was performed in a UV Stratalinker (Stratagene, La Jolla, CA) in a 24 well plate and then media and cells were transferred to a 96 well plate for MTS analysis after 48 hrs. An MTS Assay was performed after 24 hours incubation according to the manufacturer's (Promega, Madison, WI) recommendations. For long-term assays of cell survival/growth, 1 million cells expressing GFP control or FADD-DD were treated with TRAIL or etoposide for 24 hours, then washed and replaced into growth media for 7 days. Cell growth was determined by counting viable cells; the ability of surviving cells to grow was demonstrated by an increase in the total number of cells.

### Immunoblotting

Cells (1×10^6^) were harvested and lysates were prepared by boiling in SDS buffer 5 min prior to gel electrophoresis. Lysates were resolved on 12% SDS-polyacrylamide gels. Proteins were transferred to Immobilon-P Transfer Membrane (Millipore Corporation, Bedford, MA). Blots were blocked with 5% milk in TBST and incubated with antibodies that recognize IKappaB-alpha, phospho-JNK, JNK, Caspase 8, Caspase 3 (Cell Signaling Technologies, Danvers, MA), FADD (BD Biosciences, Franklin Lakes, NJ). Blots were then incubated with anti-rabbit or anti-mouse horseradish peroxidase-conjugated secondary antibodies (Cell Signaling Technologies, Danvers, MA). Detection was performed using chemiluminescent ECL reagent (Millipore Corporation, Bedford, MA) and developed on Blue X-Ray film (Life Science Products, Inc., Frederick, CO).

### Transfection of HCT116

Cells (1×10^6^) were plated in a 6 well dish and transfected with GFP, GFP-FADD-DD, or GFP-FADD-DD V108E using Lipofectamine 2000 (Life Technologies Corporation, Carlsbad, CA). Transfection efficiency was verified using fluorescent microscope 24 hrs after transfection. Cells were trypsinized and replated at 16,000 cells per well in a 96 well plate and then allowed to sit down overnight. Cell death assays were then conducted.

### Tumor Treatment studies

Groups of 3–4 NOD/SCID mice were subcutaneously injected at two sites/mouse with 1×10^7^ BJAB cells and tumors allowed to grow to a size of ∼200 mm^3^ prior to randomization into control or treatment (IP injection of etoposide (15 mg/kg twice a week) groups. Tumor size was monitored every other day using vernier digital calipers in three dimensions and calculated as a spheroid tumor volume (h×w×l×0.523). Tumor growth in the treated animals was compared between groups using t-test. For tumor western blotting, Paraffin-embedded tumors were deparaffinized in xylene, rehydrated in graded ethanol, immersed in distilled water, and air-dried. Tumors were diced into small pieces and homogenized in RIPA buffer containing 2% SDS. Samples were heated at 100°C for 20 min and then incubated at 60°C for 2 hrs. Debris was centrifuged twice to leave the supernatant for western blotting.
